# Mercury Adsorption and Oxidation Performance of an Iron-Based Oxygen Carrier during Coal Chemical Looping Process

**DOI:** 10.3390/molecules29102195

**Published:** 2024-05-08

**Authors:** Guochao Hu, Shuju Zhao, Minggang Gao, Yongzhuo Liu

**Affiliations:** Laboratory of Green & Smart Chemical Engineering in Universities of Shandong, College of Chemical Engineering, Qingdao University of Science and Technology, Qingdao 266042, China; huguochao1008@163.com (G.H.); zhaoshuju1015@163.com (S.Z.); gaominggangqust@163.com (M.G.)

**Keywords:** chemical looping combustion, chemical looping gasification, mercury, coal, iron-oxygen carrier

## Abstract

During chemical looping combustion (CLC) and chemical looping gasification (CLG) of coal, the release, migration, and speciation of mercury in coal are significantly influenced by oxygen-carrier materials; however, the underlying mechanism remains inadequately addressed. In this work, the effect of a typical iron-based oxygen carrier on the release behavior of mercury from a bituminous coal and a lignite was investigated based on the Ontario-Hydro method. It is found that the effect of the iron-based oxygen carrier is attributed to three aspects: the enhanced release rate of mercury from coal, the adsorption of the released mercury, and the oxidization of gaseous Hg^0^ into Hg^2+^. With the increasing temperature, the adsorbance of mercury by the iron-based oxygen carrier decreases, while the oxidation of mercury enhances. Even at 900 °C, the adsorbance of mercury by the oxygen carrier remained at 0.1687 g/g, with a relative content of Hg^2+^ at 22.55%. Additionally, it was observed that iron-based oxygen carriers can physically absorb both Hg^0^ and Hg^2+^, while chemisorption refers to complex-compound formation between the iron-based oxygen carrier and mercury.

## 1. Introduction

Mercury has been classified as a global pollutant, along with greenhouse gases, by United Nations Environment Programme (UNEP), due to its high volatility, toxicity, and bioaccumulation. Every year, about one third of the global total mercury emissions comes from coal combustion. As a result, there is an increasing focus on controlling mercury emissions from coal combustion worldwide [1]. In traditional coal combustion and gasification processes, three mercury species are ultimately generated—gaseous elemental mercury Hg^0^ (g), gaseous oxidized mercury Hg^2+^ (g), and particle-bound mercury Hg (p)—after a series of complex reactions [2,3,4]. However, each mercury form possesses distinct characteristics and environmental migration capabilities. Among the three mercury species, gaseous mercury Hg^0^ (g) is the most challenging to remove, while Hg^2+^ (g) and Hg (p) are more easily removable. Therefore, it is of great significance to understand the release, migration, and removal of mercury from coal.

Due to its high-energy efficiency, controllable redox degree, and inherent product separation, chemical looping has been regarded as a promising strategy for carbon capture storage (CCS) in utilizing fossil fuel, as well as a prospective approach for producing hydrogen or chemicals [5]. Over the past dozen years, extensive research has been conducted on coal chemical looping combustion (CLC), leading to several industrial demonstrations [6]. With syngas or hydrogen as target products, chemical looping gasification (CLG) of coal or biomass with less oxygen carrier for fuel has also gained significant attentions [7,8]. In comparison to traditional gasification, the oxygen carrier in CLG serves not only as a catalyst but also as an oxygen and heat carrier for gasification reactions [9], avoiding the air separation unit (ASU). However, there is limited research available on the release behavior of mercury during both CLC and CLG of coal with a different ratio of oxygen carrier to fuel.

**Figure 1:**
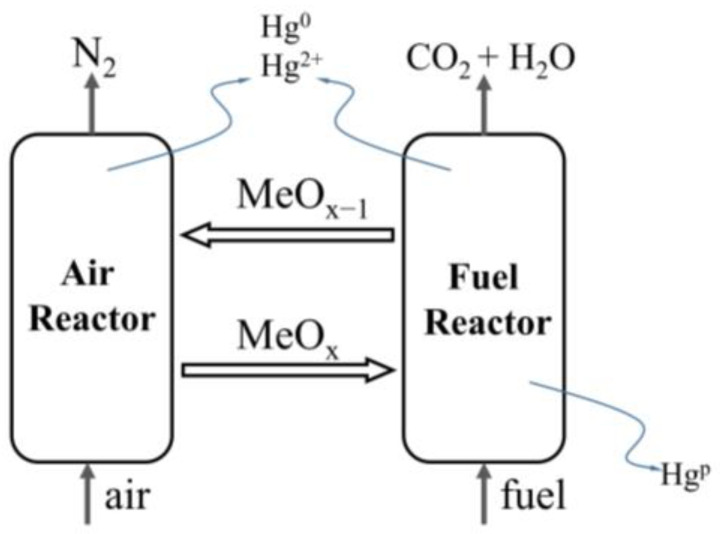
Diagram of mercury release during chemical looping process.

The schematic depiction of a potential release route of mercury during the chemical looping process is depicted in Figure 1. The chemical looping process involves two separated reactors interconnected by an oxygen carrier. In the fuel reactor, the reaction occurs between coal and an oxygen carrier in the presence of a gasified agent. Simultaneously, mercury is released from coal in the form of gaseous mercury and particulate mercury that is fixed on fly ash, slag, or an oxygen carrier. Accompanying the reduced oxygen carrier and unburned coal, unreleased mercury is transported to the air reactor, where it is released at a high temperature. Consequently, there are differences in both the release and the speciation of mercury in coal compared to traditional combustion or gasification processes [10,11]. Furthermore, variations in the reaction atmosphere and varying quantities of metal oxides during CLC and CLG can yield distinct effects on mercury-release behavior. Until now, there have been limited reports on the speciation of mercury during both CLC and CLG, particularly regarding the role played by an oxygen carrier.

As for coal CLC, Mendiara et al. [12,13] initially reported the behavior of mercury release from anthracite and lignite in an inter-connected fluidized bed using iron ore as the oxygen carrier. It was observed that 25% Hg^2+^ and 75% Hg^0^ were released from the fuel reactor while 54% Hg^2+^ and 46% Hg^0^ were released from the air reactor. The ratio of Hg^0^/Hg^2+^ mainly depends on the type of coal and FR temperature. Pérez et al. [14] investigated the release behavior of mercury in the chemical looping oxygen uncoupling (CLOU) of coal, revealing that circulating solids such as carbon, ash, and an oxygen carrier contained a mercury content of 59.4%. Ma et al. [15] reported that the hematite oxygen carrier promoted the conversion of Hg^0^(g) to Hg^2+^(g). Regarding the coal CLG process, An et al. [16,17] reported that a CuFe_2_O_4_ oxygen carrier promoted the transformation of Hg (g) to Hg (p) and gaseous oxidized mercury (Hg^2+^), and revealed the effect of the oxygen vacancy structure of CuFe_2_O_4_ oxygen carrier on the reaction between H_2_S and Hg^0^.

As an oxygen and heat carrier between two reactors, the oxygen carrier not only participates in redox reactions in both reactors, but also undergoes recycling between them. The effect of the oxygen carrier on the release, migration, and speciation of mercury in the coal is inevitable. Ghorishi et al. [18] pointed out that the unburned carbon and some active inorganic chemical components (such as Fe_2_O_3_, CuO and MnO_2_) present in fly ash from coal-fired power plants play a significant role in promoting the oxidation of elemental mercury Hg^0^ (g). Galbreath et al. [19] discovered that α-Fe_2_O_3_, γ-Fe_2_O_3_, and HCl promoted the transformation of (Hg^0^) to (Hg^2+^) in the flue gas emitted by coal burning. Recently, Ni et al. [20] ranked different oxygen carriers for elemental mercury oxidation based on their Cl/Cl_2_ ratios for CLC purposes. The results demonstrated that the order of effectiveness for various oxygen carriers was as follows: CaSO_4_ > Co_3_O_4_ (Mn_2_O_3_) > Fe_2_O_3_ > CuO (CeO_2_) > SiO_2_ (Al_2_O_3_).

Additionally, due to its porous nature, an oxygen carrier exhibits excellent mercury-adsorption capabilities. Li et al. [21] and Zhang et al. [22] investigated the mercury adsorption on various crystal surfaces of Fe_2_O_3_ at high temperatures, revealing that the adsorption process involves both physical adsorption and chemisorption between Fe_2_O_3_ oxygen carriers and Hg. The calculation results of Guo et al. [23] demonstrated that the formation of hybrid orbital between Fe and Hg contributes to the strong adsorption of mercury by γ-Fe_2_O_3_. Furthermore, the calculation of Jung et al. [24] indicated that the presence of Cl enhances the adsorption strength between Hg and α-Fe_2_O_3_.

In this work, the release behavior of mercury in both CLC and CLG with a prepared iron-based oxygen carrier and two typical coals (JYC bituminous coal and ZTC lignite coal) was investigated. Additionally, the adsorption and oxidation of mercury by the iron-based oxygen carrier were confirmed.

## 2. Results and Discussion

### 2.1. Effect of Oxygen Carrier on Release Behavior of Mercury in Coal

**Figure 2:**
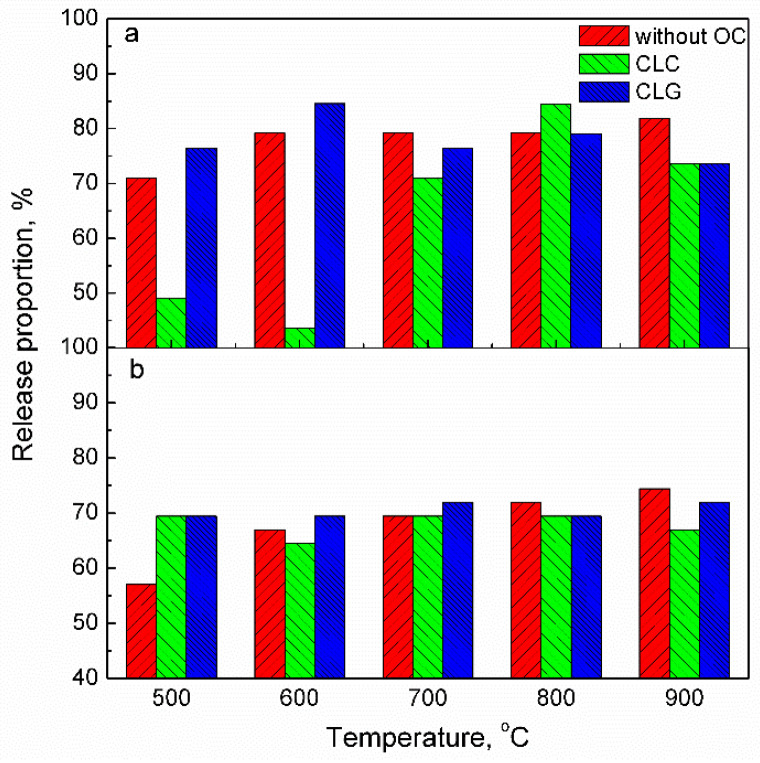
Release proportion of mercury from fuel reactor without and with iron-based oxygen carrier (**a**) JYC; (**b**) ZTC.

The release proportion of mercury from coal is significantly influenced by temperature. Aiming to reveal the effect of an oxygen carrier on the release behavior of mercury, five temperatures and two coals (JYC bituminous coal and ZTC lignite coal) without or with an oxygen carrier were investigated. As illustrated in Figure 2, in the absence of an oxygen carrier, the release proportion of mercury increases with the increasing temperature for both types of coal. For JYC bituminous coal, it increases from 70.96% at 500 °C to 81.88% at 900 °C while for ZTC lignite coal it increases from 57.04% at 500 °C to 74.4% at 900 °C. In contrast, when using an iron-based oxygen carrier, there is a significant effect on the release proportion of mercury. Specifically, for JYC coal (as observed in Figure 2a), it can be seen that the presence of an iron-based oxygen carrier restrains mercury release except at a temperature of 800 °C in CLC; however, it initially improves and then suppresses mercury release in CLG.

The release of mercury can be enhanced through the catalytic effect of an oxygen carrier, while it can be mitigated by the adsorption of an oxygen carrier. Firstly, an oxygen carrier serves as a catalyst for coal pyrolysis, gasification, and combustion processes. Consequently, it accelerates the release rate of mercury from coal with increasing temperature. On the other hand, as a porous material, the oxygen carrier can physico-chemically adsorb the released mercury. For instance, at 500 °C without an oxygen carrier, JYC coal exhibits a release proportion of 70.96%, whereas in CLC and CLG systems, this proportion accounts for 49.09% and 76.37%, respectively. The presence of an oxygen carrier significantly suppresses mercury release in CLC while promoting its release in CLG. As the amount of oxygen carrier used in CLC is twice that used in CLG for the same amount of coal, its adsorption capability outweighs its acceleration effect on release rates during the CLC process. At 900 °C, the lower proportion of mercury released is likely attributed to chemisorption by forming new substances.

The effect of the oxygen carrier on mercury release from ZTC lignite, as depicted in Figure 2b, exhibits a similarity to that of JYC. However, the enhancement caused by the oxygen carrier is more pronounced. Even at a temperature of 500 °C in CLC, the proportion of mercury release increases from 57.04% to 69.44%. Furthermore, it is noteworthy that temperature has a significant influence on mercury release. The proportion of mercury released rises from 57.04% at 500 °C to 74.4% at 900 °C. The disparity between these two types of coals can be attributed to their distinct structures and compositions. The release of mercury is more pronounced in low-rank coal.

**Figure 3:**
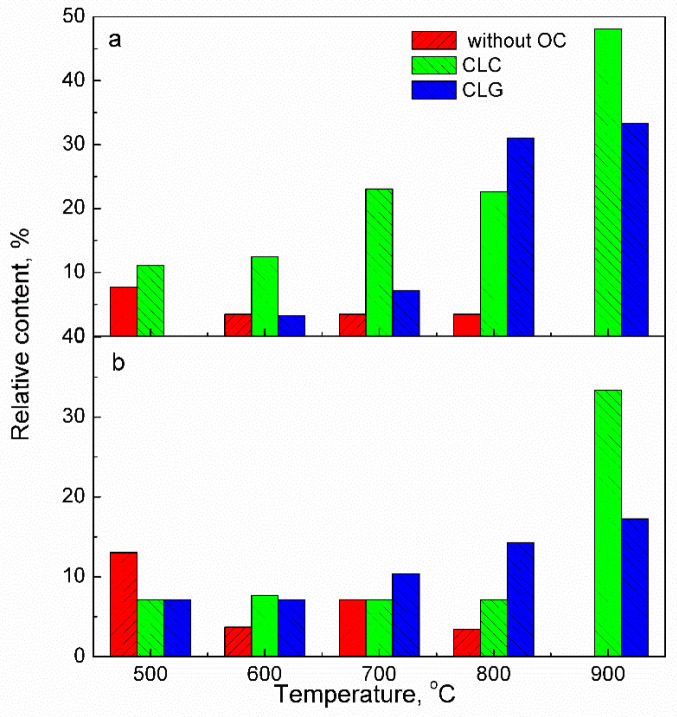
Relative content of Hg^2+^ in releasing gaseous mercury from fuel reactor at different temperature without and with iron-based oxygen carrier (**a**) JYC; (**b**) ZTC.

The release of gaseous mercury as a function of temperature was further investigated, with particular focus on the relative content of Hg^2+^. As depicted in Figure 3, in the absence of an oxygen carrier, the relative content of Hg^2+^ gradually decreases with increasing temperature until it reaches zero at 900 °C. In contrast, the presence of an iron-based oxygen carrier enhances the relative content of Hg^2+^ as temperature increases in both CLC and CLG processes. This phenomenon is contrary to traditional coal gasification or combustion processes where Hg^0^ is dominant. The chemical looping process for JYC coal is illustrated in Figure 3a. It can be observed that the increase in Hg^2+^ concentration is higher in CLC compared to CLG at all temperatures except for 800 °C. At 900 °C, the maximum relative content of Hg^2+^ reaches 48.15% in CLC and up to 33.34% in CLG due to the high oxygen carrier-to-coal mass ratio employed in CLC. Figure 3b shows the release profile of Hg^2+^ from ZTC, which exhibits a different structure and composition compared to JYC coal. In this case, at a temperature of 900 °C, the maximum relative content of Hg^2+^ reaches up to 33.33% in CLC and up to17.24% in CLG. In all cases, the iron-based oxygen carrier demonstrated excellent oxidation of mercury.

**Figure 4:**
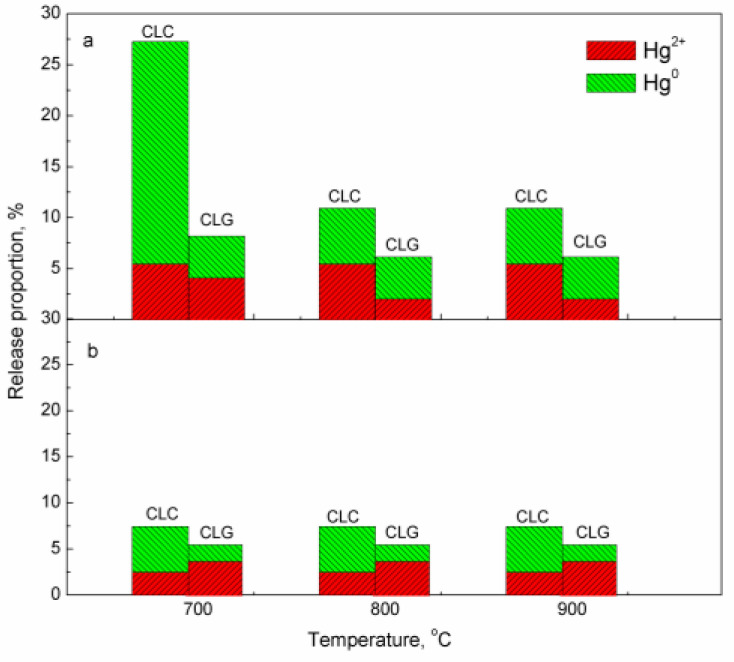
Release proportion of Hg^0^ and Hg^2+^ from air reactor (**a**) JYC; (**b**) ZTC.

When the reaction atmosphere was switched to air, both the adsorbed mercury by the oxygen carrier and the unreleased mercury from coal were released in the air reactor. The release proportions of Hg^0^ and Hg^2+^ from the air reactor are shown in Figure 4. As for JYC, the release of total gaseous mercury in the air reactor of both chemical looping processes decreases as the temperature of the fuel reactor increases, which can be attributed to an increase in mercury release in the fuel reactor with the elevating temperature. This finding is consistent with the results presented in Figure 2. Furthermore, owing to the oxidation of molecule oxygen in air, a portion of gaseous mercury is released in the form of Hg^2+^. Nevertheless, the proportion of Hg^2+^ released in the air reactor is lower that of Hg^0^, except for the CLG of ZTC coal. Additionally, there is a constant release of total gaseous mercury and Hg^2+^ observed between 700 and 900 °C for ZTC coal, resulting from the better reactivity of lignite coal.

Moreover, the mercury balance was calculated based on the total mercury in coal. According to the release proportion of the gaseous mercury, coals both without or with an oxygen carrier at different temperatures are within an acceptable range (70~130%) [25].

### 2.2. Adsorption of Mercury Vapor by Iron-Based Oxygen Carrier

Based on the aforementioned findings, it can be inferred that the influence of an oxygen carrier on mercury-release behavior during the chemical looping conversion of coal stems from three key factors: firstly, the acceleration of the mercury-release rate from coal; secondly, the adsorption of mercury onto the oxygen carrier; and thirdly, the oxidation of the oxygen carrier resulting in gaseous mercury formation. To validate both the adsorption and oxidation processes between the oxygen carrier and mercury, an adsorption experiment employing a mercury permeation tube was conducted.

**Figure 5:**
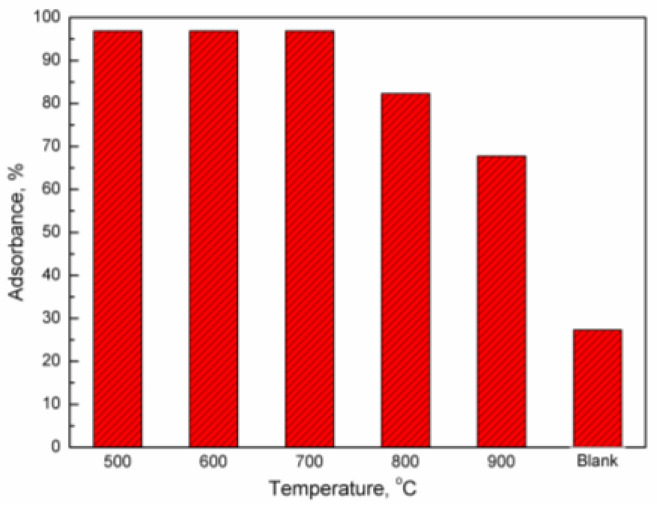
Adsorbance of mercury on iron-based oxygen carrier at different temperatures.

Figure 5 shows the adsorbance of mercury on an iron-based oxygen carrier at different temperatures. It is evident that the oxygen carrier exhibits significant adsorption on mercury vapor, with decreasing adsorbance observed as temperature increases. At 500 °C, the adsorbance of mercury on the oxygen carrier reaches 0.35 g/g, accounting for approximately 97.5% of the total mercury vapor. Even at 900 °C, a substantial proportion (67.64%) corresponding to an adsorbance value of 0.1687 g/g was still achieved by this system compared to a mere 27.30% when quartz sand was employed as a substitute for an oxygen carrier in control experiments. These results unequivocally demonstrate excellent performance exhibited by our chosen oxygen carrier in terms of its remarkable ability to effectively capture and retain elemental mercury.

### 2.3. Oxidation of Mercury Vapor by Iron-Based Oxygen Carrier

**Figure 6:**
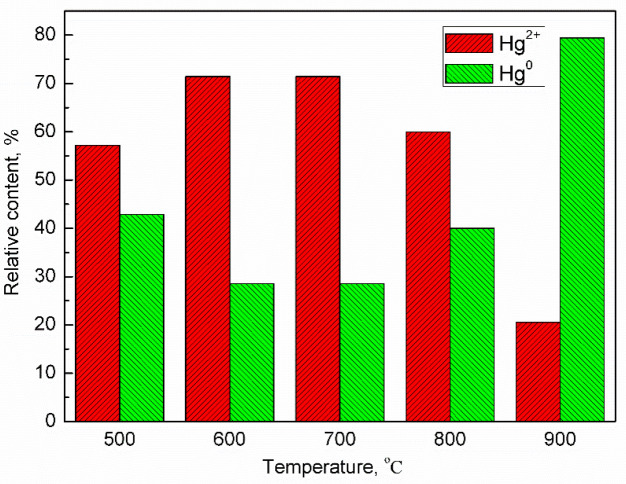
Effect of iron-based oxygen carrier on species distribution of mercury at different temperatures.

The mercury species emitted from the adsorption reactor was analyzed using Ontario-Hydro method. The distribution of mercury species at different temperatures is illustrated in Figure 6. It is evident that mercury vapor can be easily oxidized to Hg^2+^ upon passing through the oxygen carrier. However, the relative content of Hg^2+^ decreases with the increasing temperature, while that of Hg^0^ increases. At 600 °C, Hg^2+^ accounts for 71.42% of the emitting mercury from the adsorption reactor, whereas Hg^0^ accounts for 28.57%. By contrast, at 900 °C, the relative content of Hg^2+^ is reduced to only 22.55%, while that of Hg^0^ increases to 79.45%. This observation appears contradictory to the notion that oxidation of iron-based oxygen carriers is enhanced at higher temperatures; nevertheless, it verifies that there is an increase in the absolute amount of Hg^2+^ with rising temperatures due to decreased adsorbance, as shown in Figure 5.

**Figure 7:**
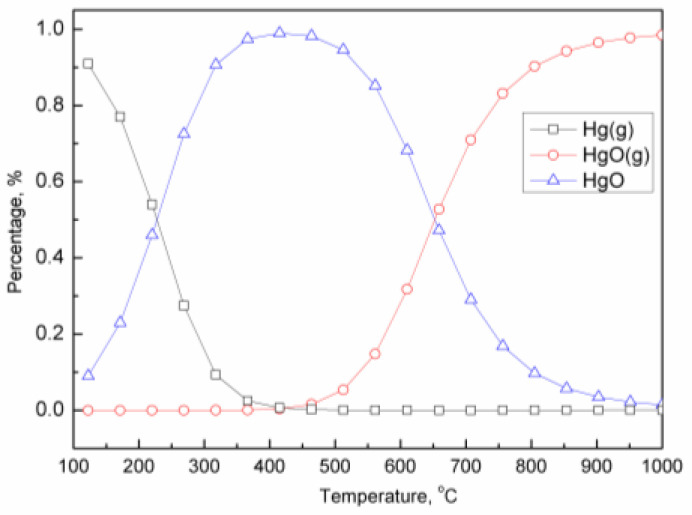
Thermodynamic analysis of species distribution of mercury in the presence of oxygen carrier.

Furthermore, the thermodynamic calculation of an Hg-iron oxygen carrier was conducted based on Gibbs free energy minimization. As depicted in Figure 7, the relative content of Hg^0^ declines to zero when temperatures exceed 400 °C, while that of HgO (g) increases close to 100%. It is widely acknowledged that the decomposition temperature of gaseous HgO is 500 °C. However, the presence of an iron-based oxygen carrier effectively inhibits the decomposition reaction of HgO.

### 2.4. Effect Mechanism of Iron-Based Oxygen Carrier on Mercury

**Figure 8:**
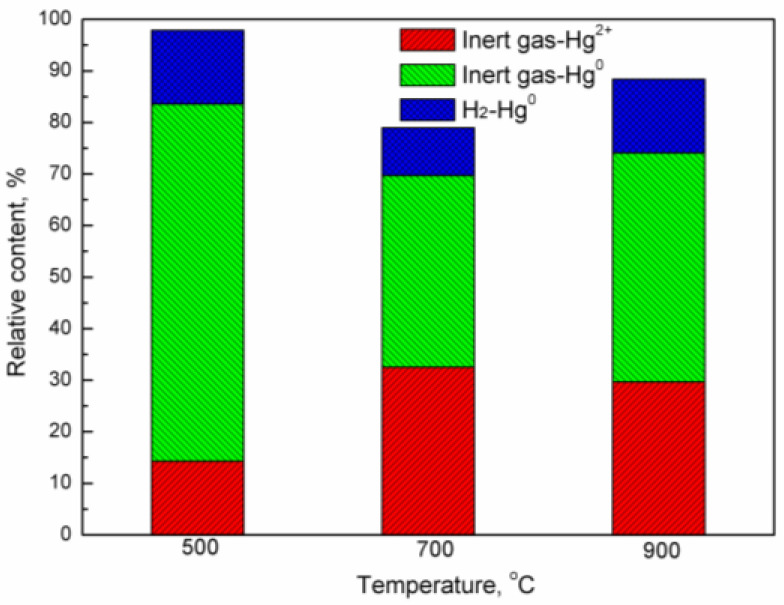
Desorption of mercury from iron-based oxygen carrier by inert and reducing atmosphere at different temperature.

In order to investigate the adsorption mechanism of an iron-based oxygen carrier on mercury, a comprehensive desorption experiment was conducted. After the cessation of mercury vapor, the iron-based oxygen carrier adsorbed with mercury was purged using inert gas for a duration of 30 min, followed by an additional purge with hydrogen at the same temperature for another 30 min. The relative content of desorbed mercury from the iron-based oxygen carrier under different temperatures and atmospheres (inert gas and reducing atmosphere) is presented in Figure 8. It is observed that more than 70% of mercury adsorbed on the oxygen carrier is desorbed after being purged by inert gas, while over 10% of the mercury is desorbed after being purged by H_2_. The desorption process involving inert gas can be attributed to physical absorption, whereas chemisorption can be ascribed to H_2_-induced desorption. Furthermore, an investigation into various forms of absorbed mercury species revealed that Hg^2+^ accounts for approximately 14.29% of released mercury at 500 °C; this value increases to 32.53% at 700 °C and then decreases slightly to 29.64% at 900 °C, respectively. These findings suggest that physically adsorbed mercury encompasses both Hg^0^ and Hg^2+^, while chemisorption may result from complex-compound formation between mercury and the iron-based oxygen carrier.

**Figure 9:**
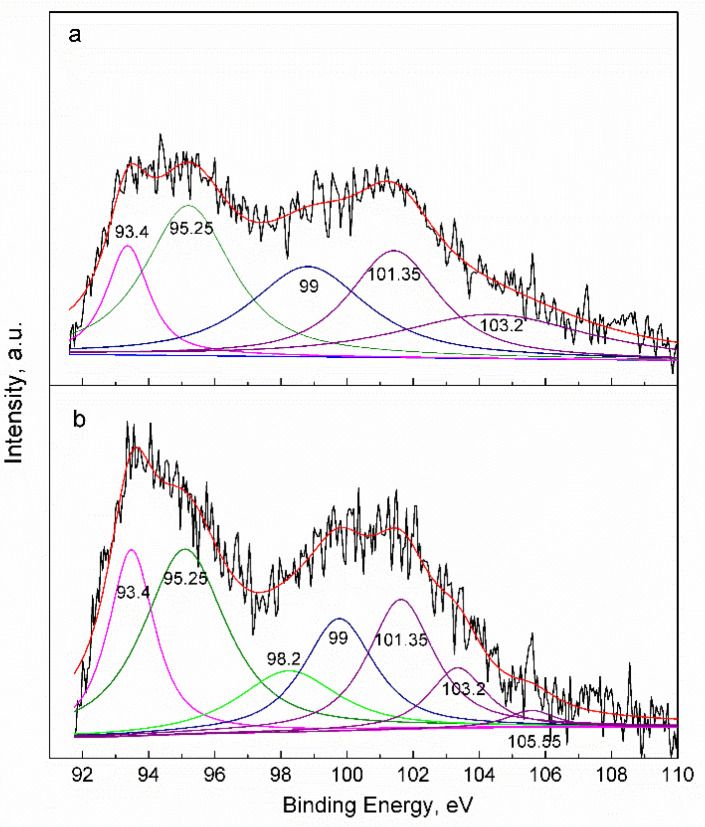
XPS spectra of iron-based oxygen carrier over the spectral regions of Hg 4f. (**a**) 500 °C; (**b**) 900 °C.

Furthermore, the oxygen carrier was subjected to XPS characterization after high-temperature mercury adsorption, as depicted in Figure 9. It is evident that the Hg4f binding energy can be resolved into five different peaks at 500 °C (Figure 9a). Among them, the binding energy of 99 and 101.5 is Hg^0^, Hg^2+^, respectively. However, by comparison with the literature, the origins of three peaks at Hg4f binding energy of 93.4, 95.2, and 103.3 remain unknown. Based on these experimental findings, it is plausible to suggest that these peaks may arise from chemisorption processes between the iron-based oxygen carrier and mercury, leading to complex-compound formation with the oxygen carrier itself. Similarly, at a higher temperature of 900 °C (Figure 9b), the Hg 4f binding energy exhibits seven discernible peaks, which are also attributed to complex-compound formation between mercury and the iron-based oxygen carrier.

## 3. Experimental and Methods

### 3.1. Preparation and Characterization of Materials

According with our previous work [26,27], a Fe_4_Al_6_ oxygen carrier was prepared with the mass ratio of Fe_2_O_3_ to Al_2_O_3_ equal to 4:6. The detailed procedure is as follows: the stoichiometric quantities of ionic nitrates Fe (NO_3_)_3_·9H_2_O were dissolved in distilled water to form a saturated solution. Subsequently, Al_2_O_3_ was slowly added into the solutions while stirring. The obtained viscous material was dried and calcined in a muffle furnace at 900 °C for 3 h. Then, the calcined materials were cooled, crushed, and sieved for use. Due to the analytical purity of the raw material, no other impurities, including mercury, were present.

As for coal, two kinds of coal, including a bituminous coal (JYC) and a lignite coal (ZTC, Suzhou, China), were adopted in this work. After being dried and grinded, the basic properties, including ultimate analysis, proximate analysis, and mercury content, were analyzed (Table 1), where the mercury contents of JYC and ZTC were 0.229 μg·g^−1^ and 0.252 μg·g^−1^, respectively. The ultimate analysis was conducted using an elemental analyzer (Elementar UNICUBE, Langenselbold, Germany), with oxygen content determined by the difference method. Proximate analysis was performed using a thermogravimetric analyzer (NETZSCH STA409PC). Mercury content was examined via ICP-MS (Agilent 8900, Santa Clara, CA, USA)

### 3.2. Experimental Setup and Procedure

**Figure 10:**
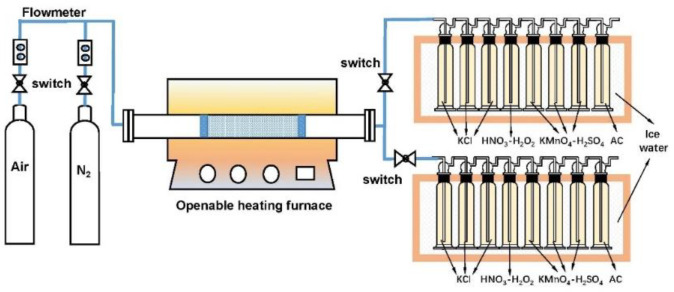
Schematic diagram of experimental system.

As illustrated in Figure 10, the experimental system is composed of an openable heating furnace (OTF-1200X, Hefei Kejing, Hefei, China), a gas distribution system, a gaseous mercury absorption device (including a train of impingers), a digestion device (DigiBlock, Beijing Labtech, Beijing, China), and a mercury content determination device (CVAAS, F732-VJ, Shanghai Huaguang, detection range 0~10 g/L; limit of detection ≤ 0.05 g/L; linear error ± 10%; repetitive ≤ 3%). According to the Ontario-Hydro method [28], the gaseous mercury absorption device includes eight successively connected impingers with the volume 125 mL. The first three impingers with 1 mol/L KCl solution are used for absorbing Hg^2+^, the following four impingers are used for absorbing Hg^0^, with 5% (*v*/*v*) HNO_3_-10% (*v*/*v*) H_2_O_2_ solution in the fourth impinger and 4% (*w*/*v*) KMnO_4_-10% (*v*/*v*)-H_2_SO_4_ solution in other three impingers. The eighth impinger, filled with activated carbon (AC), is adopted to absorb mercury vapor to prevent environmental pollution. Two trains of impingers were used, alternately, for adsorbing the gaseous mercury from the fuel reactor and the air reactor, simulated by switching the reaction atmosphere. The detailed approaches, including solution preparation, digestion method, and reagents, were performed according to the Ontario-Hydro method.

In order to investigate the release behavior of mercury in coal, both the proportion of the released mercury and the mercury species was studied. The detailed experimental procedure is as follows. For each test, a mixture of 4 ± 0.0001 g coal and the corresponding iron-based oxygen carrier was filled into tube reactor, with the amount of oxygen carrier determined based on the molar ratio of releasable lattice oxygen in the oxygen carrier to carbon content in coal [29]. The mass of the oxygen carrier was determined as 200 g, 100 g YJC coal for CLC and CLG, respectively, while that for ZTC coal was 95.2 g, 47.6 g in CLC and CLG, respectively. Ceramic porous baffle was placed at both end of the tube reactor to prevent any blowout of mixtures. After linking each device without air leakage, the experimental system was purged with high-purity inert gas at a flow rate of 100 mL/min. Once the specified temperature (500 °C, 600 °C, 700 °C, 800 °C, 900 °C) was obtained by the tube furnace, a quartz tube containing the mixed material was quickly placed in its constant temperature section. Simultaneously, one train of impingers absorbed the gaseous mercury, including Hg^0^ and Hg^2+^, for a duration of 30 min. Subsequently, the temperature was raised to 900 °C, followed by switching to air atmosphere with the flow 200 mL/min. At the same time, the other train of impingers absorbed the other gaseous mercury for 40 min. Finally, the absorbents were filled to the specific volume, recovered, digested, and analyzed, referring to the Ontario-Hydro method. Every experiment was repeated several times. Additionally, the analysis of absorbents was repeated and averaged.

In order to validate the mercury adsorption and oxidation properties of the oxygen carrier, a system reconstruction was conducted for the adsorption experiment. A mercury generator equipped with a mercury permeation tube (VICI) was utilized. Subsequently, 50 g oxygen carrier, or Al_2_O_3_ for blank experiment, was filled into a quartz tube reactor. After ensuring no air leakage by connecting all devices, the experimental system was purged with high-purity inert gas at a flow rate of 100 mL/min. Once it had reached the specified temperature (500 °C, 600 °C, 700 °C, 800 °C, 900 °C), the mercury generator was activated at a generating rate of 188 ± 17 ng·min^−1^. Following a reaction time of 30 min, the mercury generator was turned off. The oxygen carrier was then cooled down to room temperature and stored for XPS analysis to determinate any adsorbed species using CVAAS.

The desorption experiment is similar to the adsorption experiment. After shutting down the mercury generator, an additional purge period of another 30 min was performed on the oxygen carrier while simultaneously absorbing this purge gas using one train of impingers. Subsequently, hydrogen gas with a flow rate of 100 mL/min from a hydrogen generator replaced the purge gas for another duration of 30 min. Finally, CVAAS analysis determined any adsorbed species of mercury.

### 3.3. Data Evaluation

(1) Release proportion of mercury is defined as

(1) $$\eta =\frac{m_{{Hg}^{0}}+m_{{Hg}^{2+}}}{m_{{Hg}_{total}}}$$

where $m_{{Hg}^{0}}$ and $m_{{Hg}^{2+}}$ is the weight of Hg0 in gaseous mercury obtained using the Ontario-Hydro method; $m_{{Hg}_{total}}$ is the weight of total mercury in coal added to reactor.

(2) Relative content of Hg^2+^ is defined by

(2) $$\omega =\frac{m_{{Hg}^{2+}}}{m_{{Hg}^{0}}+m_{{Hg}^{2+}}}$$

## 4. Conclusions

In this work, the mercury adsorption and oxidation properties of an iron-based oxygen carrier were observed during CLC and CLG of coal, which was further confirmed by adsorption–desorption experiments with mercury vapor. The following conclusions can be drawn,

(1) The effect of an iron-based oxygen carrier on mercury release in chemical looping conversion is attributed to three aspects: the enhanced release rate of mercury from coal, the adsorption of mercury on the surface of the oxygen carrier, and the oxidation of gaseous mercury from Hg^0^ to Hg^2+^.
(2) With the increasing temperature, the adsorbance of mercury by the iron-based oxygen carrier decreases, while its oxidation of mercury enhances. Even at 900 °C, the adsorbance of mercury by the oxygen carrier remained at 0.1687 g/g, with a relative content of Hg^2+^ at 22.55%.
(3) The mercury adsorption on the surface of the iron-based oxygen carrier involves both chemisorption and physical absorption. Physical adsorption includes both Hg^0^ and Hg^2+^, while chemisorption refers to complex-compound formation between mercury and the iron-based oxygen carrier.

## Figures and Tables

**Table 1:** Ultimate analysis, proximate analysis, and mercury content of the used coal.

	Proximate Analysis, W_ad_/%				Ultimate Analysis, W_ad_/%					Hg, μg·g^−1^
	M	A	V	FC	C	H	O	N	S	
JYC	1.88	8.36	34.08	55.68	74.74	4.58	8.66	1.28	0.50	0.229
ZTC	22.38	23.5	30.98	23.14	35.6	2.21	14.49	0.93	0.89	0.252

M = Moisture, A = Ash, V = Volatile, FC = Fixed Carbon.

## Data Availability

The original contributions presented in the study are included in the article, further inquiries can be directed to the corresponding author.
